# RAS, a Pentatricopeptide Repeat Protein, Interacts with OsTRX z to Regulate Chloroplast Gene Transcription and RNA Processing

**DOI:** 10.3390/plants14020247

**Published:** 2025-01-16

**Authors:** Zhennan Qiu, Shiyong Wen, Peinan Sun, Dongdong Chen, Chunmiao Wang, Xiliang Song, Liying Xiao, Peiliang Zhang, Dongying Zhao, Cuiping Wen, Peiyan Guan, Xuechu Du, Yinghui Sun, Chenshan Xu, Jian Song

**Affiliations:** 1 College of Life Science, Dezhou University, Dezhou 253023, China; wenshiyong434@163.com (S.W.); fengjisunpeinan@163.com (P.S.); wangchunmiaomiao@163.com (C.W.); sxl0424@126.com (X.S.); xiao1991liying@126.com (L.X.); zplhlm@163.com (P.Z.); zhaodongying4321@163.com (D.Z.); wencuiping913@163.com (C.W.); guanpeiyan@126.com (P.G.); chwang1001@126.com (X.D.); sunyh8789@dzu.edu.cn (Y.S.); michael_10@163.com (C.X.); jsongbio@gmail.com (J.S.); 2 State Key Laboratory of Rice Biology, China National Rice Research Institute, Hangzhou 310006, China; 15757906206@163.com

**Keywords:** rice, PPR, chloroplast development, RNA splicing, RNA editing

## Abstract

Thioredoxin z (TRX z) plays a significant role in chloroplast development by regulating the transcription of chloroplast genes. In this study, we identified a pentatricopeptide repeat (PPR) protein, rice albino seedling-lethal (RAS), that interacts with OsTRX z. This interaction was initially discovered by using a yeast two-hybrid (Y2H) screening technique and was further validated through Y2H and bimolecular fluorescence complementation (BiFC) experiments. RAS contains 16 PPR motifs and features a small MutS-related (SMR) domain at its C-terminus. CRISPR/Cas9-generated *ras* mutants exhibited an albino seedling-lethal phenotype characterized by abnormal chloroplast structures and a significantly reduced chlorophyll content. RAS localizes to the chloroplast and is predominantly expressed in young leaves. Mutations in *RAS* affect RNA editing at the *rpl2*, *rps14*, and *ndhA* sites, as well as RNA splicing at the *rpl2*, *atpF*, and *ndhA* transcripts within the chloroplast. Furthermore, the expression levels of genes associated with chloroplast formation are altered in the *ras* mutant. Both OsTRX z and RAS were found to interact with chloroplast signal recognition particle (cpSRP) proteins, indicating that their proper localization within the chloroplast may be dependent on the SRP pathway. Collectively, our findings highlight the critical role of RAS in chloroplast development, as it is involved in RNA processing and the regulation of chloroplast gene expression.

## 1. Introduction

Chloroplast development is orchestrated by plastid-encoded plastid RNA polymerase (PEP) and nuclear-encoded plastid RNA polymerase (NEP) [1,2]. Thioredoxin z (TRX z), a member of the thioredoxin family, is originally identified as a component of plastid transcriptionally active chromosomes (pTACs), which consist of core PEP subunits, DNA, RNA, and some other proteins [2,3]. Additional research has indicated that TRX z is a fundamental component of the PEP complex, essential for the transcription of genes in chloroplasts [3,4]. TRX z binds to the fructokinase-like proteins FLN1 and FLN2, which are essential components of pTACs. This TRX z–FLN complex plays a role in regulating PEP activity, which subsequently influences chloroplast gene expression [3,4,5]. In addition, TRX z also interacts with the putative plastidic oxidoreductase temperature-sensitive virescent (TSV), plastid multiple organellar RNA editing factors (OsMORFs), the pentatricopeptide repeat protein seedling stage albino 1 (SSA1), and the single-stranded DNA binding protein WHIRLY1 [6,7,8,9]. Overall, TRX z is a critical regulator of chloroplast biogenesis and function. Ongoing research is essential to further elucidate the precise mechanisms by which TRX z influences chloroplast development, and to understand its place within the intricate chloroplast regulatory network.

Pentatricopeptide repeat (PPR) proteins represent one of the most extensive protein families found in terrestrial plants [10,11,12]. PPR proteins are defined by multiple consecutive repeats of a 35-amino acid motif that is crucial for their ability to bind RNA [13,14]. PPR proteins are typically divided into two main subfamilies: the P subfamily and the PLS subfamily, which is determined by the existence of extra conserved motifs [11,15]. P-type PPR proteins consist solely of standard 35-amino acid PPR motifs and primarily play roles in RNA splicing, cleavage, stabilization, and the initiation of translation [10,13,16]. Additionally, a few members of the P subfamily have an extra small MutS-related (SMR) domain at the C-terminus, which leads to their categorization as the PPR-SMR subgroup [15,17,18].

PPR proteins are essential for the post-transcriptional regulation of gene expression within plant organelles, especially in mitochondria and chloroplasts [13,19]. For instance, the P-type PPR protein OsPPR11 is crucial for the splicing of introns in chloroplast RNA and plays a vital role in the development of chloroplasts in rice [20]. On the other hand, PLS-type PPR proteins contain P, L (long, 35–36 amino acids), and S (short, 31 amino acids) motifs [13,21]. Certain PLS-type PPR proteins have extra E, E+, and DYW domains at their C-terminus, which are essential for recognizing and editing RNA [10,13]. PLS-type proteins typically play a role in C-to-U editing within mitochondria and chloroplasts, which is essential for preserving transcript stability and facilitating accurate gene expression. For example, OsPPR9, a DYW-type PPR protein, affects the editing of the *rps8*, *rpoC2*, *rps14*, and *ndhB* RNA sites in rice, which influences normal chloroplast development [22]. The P-type and PLS-type PPR proteins might have some shared roles in the regulation of RNA splicing and RNA editing. Some members from both can perform both RNA editing and RNA splicing, such as young leaf white stripe (YLWS), albino seedling-lethal (OsASL), and thylakoid assembly 8 (OsTHA8) [23,24,25]. This overlapping functionality suggests some redundancy or cooperation among these PPR proteins in regulating RNA processing in plants.

Many chloroplast-localized PPR proteins are involved in chloroplast development, and their mutations can result in defects leading to aberrant leaf development [10]. For example, whole albino leaf on chromosome 3 (WAL3) and SSA1 are localized in chloroplasts, and the loss of their function affects chloroplast development and leads to leaf albinism [8,26]. Additionally, PPR proteins regulate the expression of stress-related genes, affecting plant resistance to stresses [21,27]. For instance, natural blight leaf 3 (OsNBL3), a mitochondrial PPR protein, is implicated in responses to such stresses; the *nbl3* mutant shows lesion mimic phenotypes and enhanced resistance to fungal and bacterial pathogens, as well as salt stress [28]. PPR proteins also participate in cytoplasmic male sterility and endosperm development [13,21]. For example, *OsTMS19* encodes a mitochondrial PPR protein that is implicated in photo/thermo-sensitive genic male sterility, which is associated with the accumulation of excessive ROS [29]. The PPR protein FLOURY ENDOSPERM22 (FLO22) is important in endosperm development, as it participates in mitochondrial RNA splicing and RNA editing [16].

In this study, we identified a pentatricopeptide repeat (PPR) protein, RAS, that interacts with OsTRX z through yeast two-hybrid (Y2H) screening. Further investigations revealed that mutations in RAS affect the RNA editing and RNA splicing of chloroplast transcripts, as well as the expression of chloroplast-related genes. Furthermore, both OsTRX z and RAS functions may be regulated and assisted by an SRP-dependent pathway. Our findings provide insights into how nuclear-encoded chloroplast proteins coordinate gene expression and post-transcriptional RNA processing, ultimately influencing chloroplast development.

## 2. Results

### 2.1. OsTRX z Interacts with RAS

TRX z plays an essential role in the development of chloroplasts [3,30]. It interacts with several proteins involved in chloroplast development, including FLN1, FLN2, TSV, PRIN2, ALM1, OsMORFs, and OsWHY1 [4,5,6,7,9,31,32]. To identify new OsTRX z-interacting proteins, we performed Y2H screening and identified RAS (LOC_Os03g60910), a PPR protein.

An analysis of the sequence showed that the coding sequence (CDS) of *RAS* is 2,607 base pairs long, consisting of five exons, and it encodes a protein made up of 868 amino acids. As per the PPR database for plants (https://ppr.plantenergy.uwa.edu.au/ (accessed on 21 April 2024)), RAS contains 16 PPR motifs and a unique SMR domain at the C-terminus (Figure S1A). To explore its conservation, we performed a BLASTP query by using the RAS protein sequence and then conducted a homology analysis with DNAMAN. The comparison showed that sequences from different plant species exhibit approximately 89.9% identity to RAS (Figure S1B). These results indicate that *RAS* encodes a P-type PPR protein that is highly conserved across higher plants.

**Figure 1:**
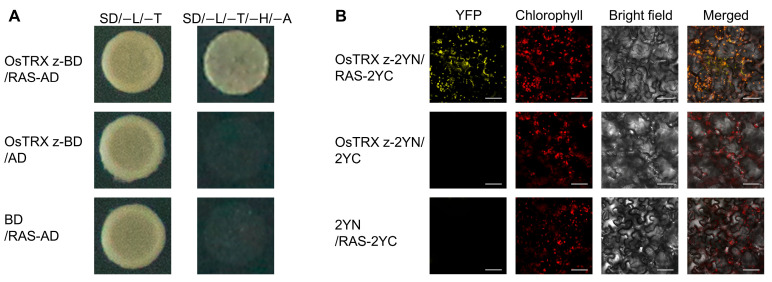
OsTRX z interacts with RAS. (**A**) Yeast two-hybrid (Y2H) assays revealed the interaction between OsTRX z and RAS. SD/−L/−T denotes a selective medium lacking leucine and tryptophan (SD/−Leu/−Trp); SD/−L/−T/−H/−A signifies a selective medium lacking leucine, tryptophan, histidine, and adenine (SD/−Leu/−Trp/−His/−Ade). (**B**) Bimolecular fluorescence complementation (BiFC) assays confirmed the interaction between OsTRX z and RAS within the chloroplasts of *Nicotiana benthamiana* leaf cells. Bar = 50 μm.

To verify that OsTRX z interacts with RAS, we performed Y2H assays. The Y2H assays indicated a potential interaction between OsTRX z and RAS proteins (Figure 1A). Yeast cells that co-expressed fusion proteins of OsTRX z and RAS showed strong growth on selective media that did not contain leucine, tryptophan, histidine, and adenine (SD/−L/−T/−H/−A), whereas the control groups failed to grow under identical conditions (Figure 1A). Bimolecular fluorescence complementation (BiFC) analysis was also employed to visualize the interaction in the chloroplast of *Nicotiana benthamiana* leaf cells. The fusion constructs, OsTRX z-2YN and RAS-2YC, were infiltrated into *Nicotiana benthamiana* leaves by *Agrobacterium* infestation. Confocal microscopy results revealed that the interaction between OsTRX z and RAS occurred specifically in the chloroplast (Figure 1B). Collectively, these findings indicate that OsTRX z interacts with RAS.

### 2.2. Characterization of the Ras Mutant

**Figure 2:**
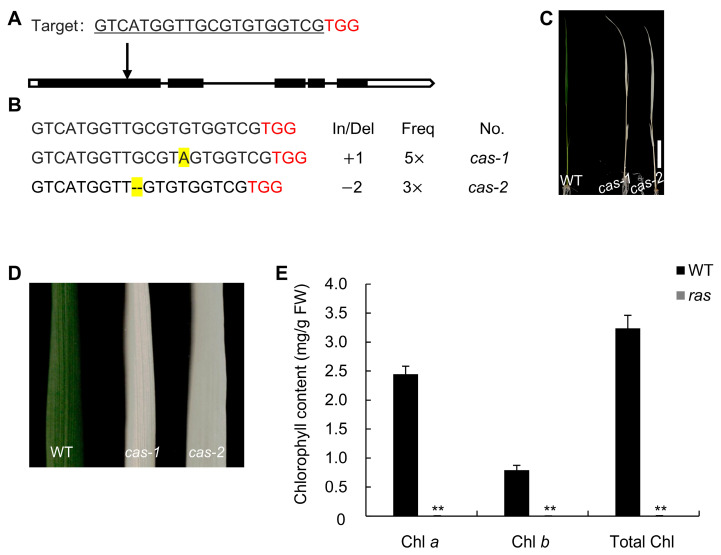
The dysfunction of *RAS* results in an albino-lethal phenotype. (**A**) Schematic of the *RAS* target site using CRISPR/Cas9. The PAM site is shown in red, and the target sequence is underlined within the first exon of *RAS*. (**B**) Two types of mutations observed at the target sequence: a 1 bp insertion and a 2 bp deletion. (**C**) Phenotypes of WT and *ras* mutants derived from gene editing. Bar = 2 cm. (**D**) Close-up image of WT and *ras* leaves. (**E**) Chlorophyll content of WT and *ras* seedlings. Chl *a*, chlorophyll *a*; Chl *b*, chlorophyll *b*. Total Chl content is the sum of chlorophyll *a* and chlorophyll *b*. The data are the mean ± SD (*n* = 3). **, *p* < 0.01 (Student’s *t*-test).

**Figure 3:**
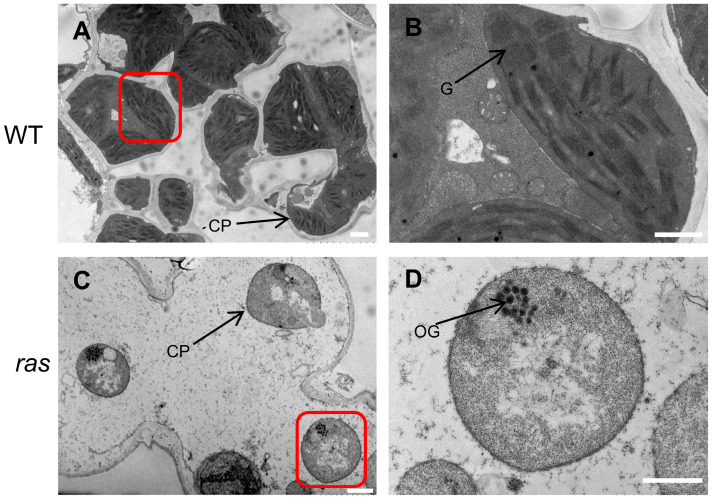
Chloroplasts in WT and *ras* leaves obtained via transmission electron microscopy (TEM). The chloroplast ultrastructure of WT (**A**,**B**) and *ras* (**C**,**D**) at the second leaf stage. The red box indicates the area magnified. CP, chloroplast; OG, osmiophilic granule; G, grana. Bar = 1 μm.

According to information from the China Rice Data Center (https://www.ricedata.cn/ (accessed on 21 April 2024)), we found that *RAS* and *OspTAC2* refer to the same gene [33]. According to earlier research highlighting the important function of OspTAC2 in the development of chloroplasts, we further investigated the function of *RAS*. To verify the functional significance of *RAS*, the pRAS-Cas9 vector was developed by utilizing CRISPR/Cas9 genome editing technology and subsequently introduced into the *japonica* rice variety Zhonghua 11 (ZH11), through *Agrobacterium*-mediated transformation. Sequence analysis showed that the heterozygous *RAS/ras* plants showed a normal phenotype. In contrast, homozygous mutants, which exhibited a 1 bp insertion and 2 bp deletion at the designated target site, manifested an albino seedling-lethal phenotype resulting from a frameshift mutation and premature translation termination (Figure 2A–C). Consistent with the albino phenotype, the chlorophyll content in the mutants was nearly undetectable (Figure 2D). Furthermore, transmission electron microscopy (TEM) revealed well-organized chloroplast structures and intact grana stacks in the leaves of the wild type (WT), whereas aberrant chloroplast structures lacking typical grana stacks were found in the *ras* mutant (Figure 3). These results indicate that the phenotype of the *ras* mutant derived from CRISPR/Cas9 is identical to that of the *osptac2* mutant derived from radiation mutagenesis.

### 2.3. Subcellular Localization and Expression Pattern Analysis

**Figure 4:**
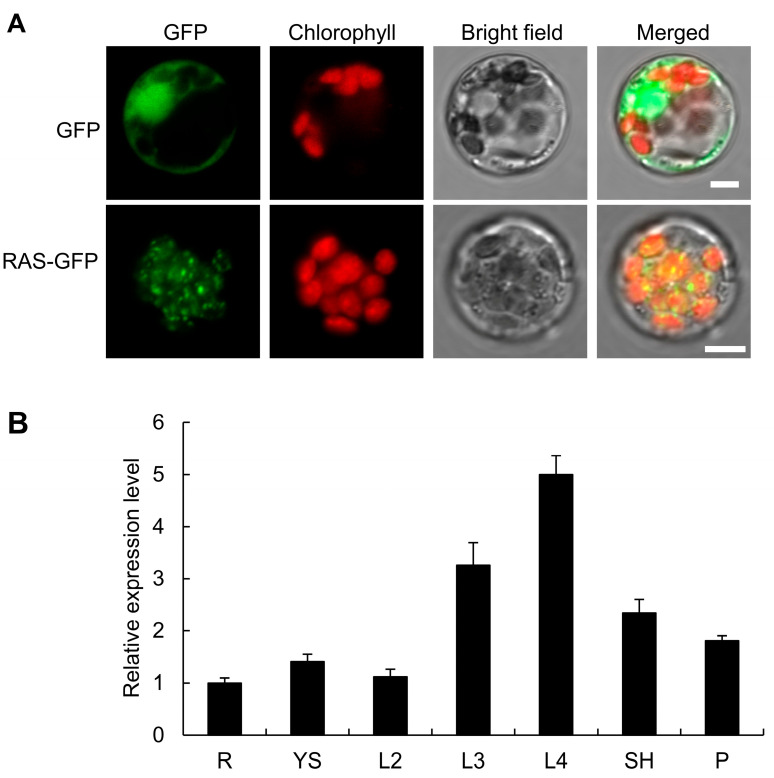
Analysis of subcellular localization and expression patterns. (**A**) Subcellular localization of the RAS protein. The subcellular localization of the RAS protein was investigated using rice protoplasts, where both a GFP control protein and a RAS-GFP fusion protein were analyzed. The green fluorescence observed corresponds to the presence of GFP, while red fluorescence indicates chloroplast autofluorescence. Additionally, yellow fluorescence signifies the presence of merged signals. Bar = 5 μm. (**B**) RAS expression in several WT seedling organs as determined by qRT-PCR. The analyzed organs include the root (R), young stem (YS), the second to fourth leaves (L2–L4), leaf sheath (SH), and panicle (P). Data are presented as mean ± SD (*n* = 3).

According to the prediction of the iPSORT (https://ipsort.hgc.jp/ (accessed on 18 May 2024)) and Wolf PSORT (https://wolfpsort.hgc.jp/ (accessed on 18 May 2024)) databases, the RAS protein is localized to the chloroplast, featuring a chloroplast transit peptide (CTP) consisting of 30 amino acids at its N-terminus. In order to experimentally confirm the subcellular localization of RAS, we constructed a plasmid, pRAS-GFP, that encodes the RAS and GFP fusion protein, utilizing the complete coding sequence of *RAS* while omitting the stop codon. Following the transformation of rice protoplasts, we noted that the green fluorescent signals exhibited co-localization with the autofluorescence produced by chloroplasts, confirming that RAS is precisely targeted to chloroplasts (Figure 4A).

To elucidate the transcriptional profile of *RAS* in WT rice plants, we performed a quantitative real-time polymerase chain reaction (qRT-PCR) analysis. The qRT-PCR data revealed the pronounced expression of *RAS* in the young leaf (Figure 4B). These findings, along with the chloroplast localization and the *ras* phenotype, support the pivotal role of RAS in chloroplast development in rice.

### 2.4. RAS Is Essential for the RNA Editing and RNA Splicing of Chloroplast Transcripts

**Figure 5:**
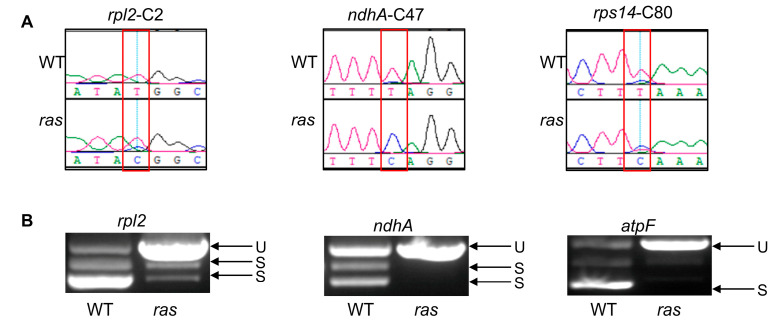
RNA editing and RNA splicing analysis. (**A**) Chloroplast RNA editing sites in WT and *ras* were sequenced. Red boxes denote non-editing sites (C) in *ras* and RNA editing sites (T) in the WT. (**B**) RNA splicing of chloroplast transcripts in WT and *ras* plants was examined using RT-PCR. S stands for spliced transcripts, and U for unspliced transcripts.

PPR proteins have been documented to play a significant role in the post-transcriptional regulation of chloroplast RNA, particularly in the mechanisms of RNA editing and RNA splicing. This involvement is illustrated by the examples of OsASL, OsTHA8, and YLWS [23,24,25]. The *RAS* gene encodes a PPR protein categorized under the P subfamily, suggesting a potential role in the RNA editing and RNA splicing of chloroplast transcripts. To determine whether RAS is involved in RNA editing, we performed sequencing and analysis of all 23 known chloroplast RNA editing sites in both WT and *ras* plants. We found that mutation in *RAS* led to defects in the editing of *ndhA*-C47, *rps14*-C80, and *rpl2*-C2 sites (Figure 5A, Table S1).

We also assessed the involvement of RAS in RNA splicing using reverse transcription PCR (RT-PCR) to analyze and compare the lengths of amplified fragments generated from primers that flank introns in WT and *ras* seedlings. The chloroplast genome of rice comprises a total of 18 introns, which consist of 1 group-I intron and 17 group-II introns [34]. Evaluations of these 18 intron sites revealed that *rpl2*, *ndhA*, and *atpF* transcripts were spliced abnormally in the *ras* mutant (Figure 5B). The splicing of other introns appeared unaffected (Figure S2).

According to the combined results, RAS is involved in both the splicing of chloroplast RNAs, such as *atpF*, *ndhA*, and *rpl2*, and the editing of chloroplast RNAs, particularly those associated with *rps14*, *rpl2*, and *ndhA*.

### 2.5. The Ras Mutant Demonstrates Modified Expression Levels of Genes Associated with Chloroplast Development

**Figure 6:**
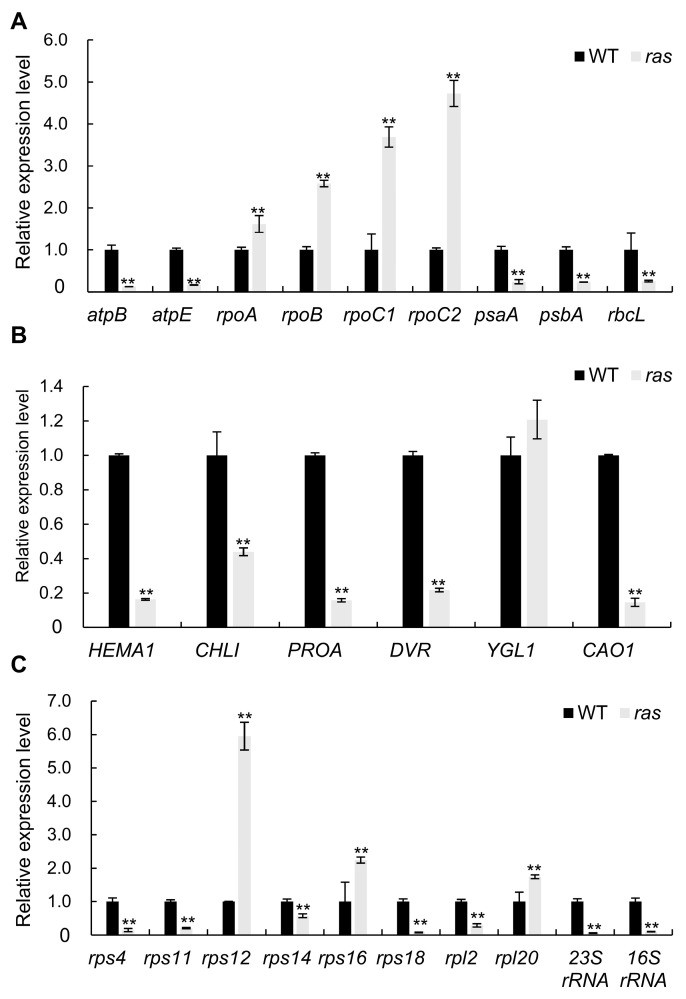
Comparative analysis of chloroplast gene expression levels in WT and *ras* seedlings. (**A**) Expression levels of plastid genes related to chloroplast development that depend on PEP-/NEP-, PEP-, and NEP. (**B**) The comparative expression levels of genes implicated in the biosynthesis of chlorophyll. (**C**) Comparative expression levels of genes associated with ribosome biogenesis. At the third leaf stage, samples were taken from WT and *ras* seedlings. Data are presented as the mean ± SD (*n* = 3). **, *p* < 0.01 (Student’s *t*-test).

To examine changes in chloroplast gene transcripts in the *ras* mutant, qRT-PCR was performed to assess the expression of chloroplast genes dependent on both NEP and PEP in WT and *ras* plants. The *ras* mutant demonstrated markedly reduced transcript levels of PEP-dependent chloroplast genes associated with photosynthesis, including *psaA*, *psbA*, and *rbc*L, in comparison to the WT (Figure 6A). Conversely, the *ras* mutant showed higher expression levels of NEP-dependent genes, including *rpoA*, *rpoB*, *rpoC1*, and *rpoC2*, than WT (Figure 6A). Furthermore, the expression levels of the NEP-/PEP-dependent genes *AtpB* and *AtpE* were significantly diminished in the *ras* mutant compared to the WT (Figure 6A).

Additionally, we examined the expression levels of genes involved in chlorophyll biosynthesis. *HEMA1*, *CHLI*, *DVR*, and *CAO1* exhibited significant reductions in the *ras* mutant compared to the WT (Figure 6B). Furthermore, the expression levels of genes associated with ribosome biogenesis were analyzed. The expression levels of *16S rRNA*, *23S rRNA*, *rps4*, *rps18*, *rps11*, *rpsl4*, and *rpl2* were significantly reduced in *ras* compared to the WT, while the expression of *rps12* and *rps16* was found to be elevated in the *ras* (Figure 6C). The results suggest that RAS plays a crucial role in regulating the expression of genes related to the formation of chloroplasts and ribosomes, as well as the production of chlorophyll.

### 2.6. OsTRX z and RAS Are Translocated to Their Destinations via an SRP-Dependent Pathway

The chloroplast signal recognition particle (cpSRP) plays a crucial role in the translocation of chloroplast proteins to the thylakoid membranes, a process that is essential for the proper development of chloroplasts [35,36]. In rice, there are two homologous cpSRPs, OscpSRP54a and OscpSRP54b, and one OscpSRP43 protein [36,37]. OscpSRP54a and OscpSRP54b exhibit the ability to interact with OscpSRP43 [36].

**Figure 7:**
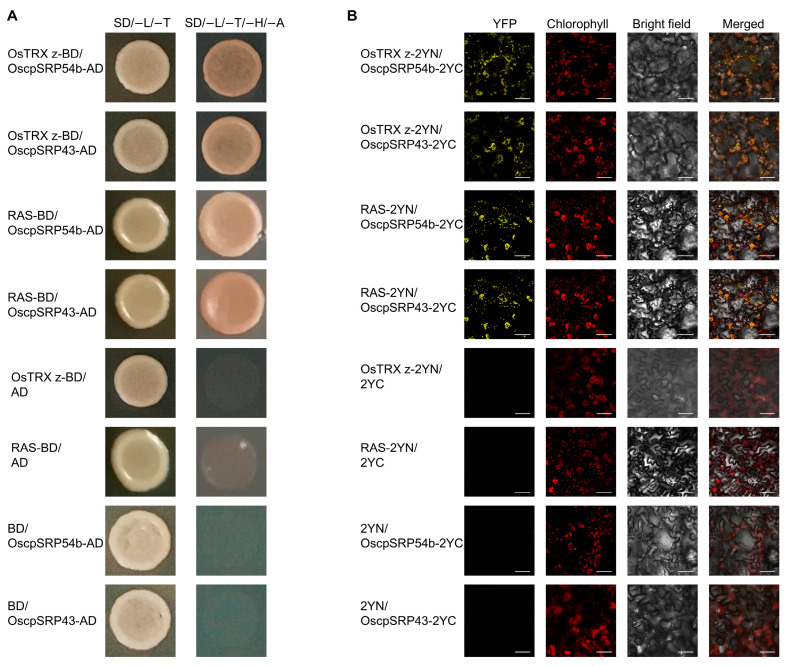
OsTRX z and RAS proteins interact with chloroplast SRP proteins. (**A**) Y2H assays showing interactions between OsTRX z and RAS with cpSRP proteins OscpSRP54b and OscpSRP43. (**B**) BiFC assays confirming interactions of OsTRX z and RAS with cpSRP proteins OscpSRP54b and OscpSRP43 in *N. benthamiana* leaves. Bar = 50 μm.

Given that OsTRX z and RAS are encoded by nuclear genes, we hypothesized that they are translated in the cytoplasm and, upon entering the chloroplast, may be transported to their destinations via the cpSRP54–cpSRP43 complex. To test this hypothesis, we conducted Y2H assays to verify the interaction between cpSRP proteins and OsTRX z or RAS. The Y2H results showed that OsTRX z and RAS could interact with OscpSRP54b and OscpSRP543 (Figure 7A). These protein interactions were further verified by using BiFC assays (Figure 7B). Together, these results indicate that OsTRX z and RAS proteins, encoded by nuclear genes, are incorporated into the cpSRP43–cpSRP54 complex and transported to their destinations by an SRP-dependent pathway after entering the chloroplast.

## 3. Discussion

It is well established that RNA polymerases encoded in the nucleus (NEP) and plastid (PEP) are both necessary for chloroplast gene transcription [38]. The PEP complex consists of two essential components: plastid-encoded core subunits and the transcriptionally active chromosomes (pTACs), which surround the PEP core subunits. pTACs stabilize the PEP structure, facilitate chloroplast gene transcription, and regulate PEP activity [39]. Earlier research has shown that TRX z is part of pTACs and works with FLN1 and FLN2 to control PEP activity during the development of chloroplasts [3,4,5,7]. In addition, TRX z interacts with other chloroplast proteins in rice, such as TSV, SSA1, and OsMORFs, suggesting that it has a multifunctional role in the development of chloroplasts [6,7,8].

In the present study, we found an interaction between OsTRX z and RAS through yeast two-hybrid (Y2H) screening, which was confirmed via both Y2H and BiFC experiments (Figure 1). Further investigation revealed that RAS is the same as the previously described OspTAC2 and contains 16 PPR motifs as well as a distinct SMR domain at the C-terminus (Figure S1A). Although the critical role of OspTAC2 in regulating chloroplast development and its effects on PEP and NEP gene expression have been established, the functional mechanisms of OspTAC2 and the broader gene regulatory network remain incompletely understood [33].

Mutations in *RAS*, generated via CRISPR/Cas9 technology, resulted in an albino seedling-lethal phenotype, accompanied by minimal chlorophyll content and abnormal chloroplast structures (Figure 2 and Figure 3). These phenotypes were consistent with previously described *osptac2* and *ostrx z* mutants [4,33]. Furthermore, *RAS* expression was primarily localized to young leaves, with the RAS protein specifically targeted to chloroplasts (Figure 4). These findings indicate that RAS is crucial in the initial stages of chloroplast formation in rice leaves during the seedling stage.

The proper expression of NEP- and PEP-related genes is critical for chloroplast development. NEP predominantly transcribes plastid housekeeping genes, including PEP core subunits such as *rpoA*, *ropB*, *rpoC1*, and *rpoC2*. These are essential for the early stages of chloroplast development. In mature chloroplasts, PEP mainly transcribes genes associated with photosynthesis, including *psaA*, *psbA*, and *rbcL* [40]. In our study, the *ras* mutant exhibited significantly reduced expression levels of PEP-related genes (Figure 6A). Conversely, NEP-related genes showed increased expression levels in the *ras* mutant (Figure 6A). The above results indicate impaired PEP activity in *ras* mutants. This upregulation of NEP-related genes may represent a feedback response to the reduced activity of PEP. Similar regulatory patterns have been observed in other rice mutants with low PEP activity, such as *oswhy1*, *ssa1*, and *wal3* [8,9,26]. Additionally, previous studies have linked reduced PEP activity to the abnormal expression of chlorophyll biosynthesis genes, which may also explain the dysregulated expression of chlorophyll synthesis-related genes observed in *ras* mutants [4,5].

PPR proteins play a role in multiple facets of organellar RNA processing, such as RNA editing and RNA splicing. RNA editing involves converting cytosine (C) into uracil (U) in mRNA, thereby modifying genetic information, while RNA splicing ensures the formation of continuous mRNA molecules essential for translation. Some PPR proteins are not only involved in RNA editing but also in RNA splicing, such as YLWS, WSL4, and OsASL [24,25,41]. In our study, we observed abnormal RNA editing at the *rpl2*, *rps14*, and *ndhA* sites, as well as abnormal RNA splicing of *rpl2*, *atpF*, and *ndhA* in the *ras* mutant (Figure 5). Previously, other rice albino mutants, such as *osppr6*, *ssa1*, and *asl* have been associated with chloroplast RNA editing abnormalities [8,25,42]; however, some studies, including those on *ostrx z* and *wsl3* mutants, suggest that seedling albinism is not always linked to defects in RNA editing [7]. According to these results, we hypothesized that defective RNA editing in *ras* may not directly cause the albino phenotype but this could instead represent a secondary effect of the mutation. Rice YLWS, WSL4, WAL3, and OsASL are involved in the splicing of plastid *rpl2*, *atpF*, and *ndhA*, and mutations in these proteins cause significant damage to chloroplast development [24,25,26,41]. In this study, RAS was also found to be implicated in the splicing of plastid *rpl2*, *atpF*, and *ndhA*. Its mutation produces the same lethal albino phenotype as observed in the *wal3* and *osasl* mutants [26].

Additionally, studies have shown that the absence of plastid ribosomal proteins, such as those encoded by *albino seedling lethal* (*asl1*), *albino lethal 1* (*al1*), and *white green leaf 2* (*wgl2*), leads to an albino seedling-lethal phenotype in rice [43,44,45]. Among genes with defective RNA splicing, *rpl2* encodes the L2 subunit of the 50S ribosomal protein, which plays an important role in the peptidyl transferase center of the plastid ribosomes. A lack of RPL2 protein serves as a sensitive indicator of damage to the ribosomal functional center. Furthermore, ribosome abnormalities and albinism are often linked to the ineffective splicing of *rpl2* in both *Arabidopsis* and rice [46,47]. In our study, defective *rpl2* splicing in the *ras* mutant appeared to cause the abnormal expression of plastid ribosomal genes, especially the reduced expression of *rpl2*, which impaired the translational apparatus and subsequently disrupted chloroplast development. According to these findings, RAS is crucial for the RNA editing and RNA splicing processes of chloroplast transcripts. We further speculate that the impaired splicing of defective *rpl2* hinders the translation of chloroplast proteins by interfering with the formation of plastid ribosomes, resulting in reduced PEP activity and abnormal development of chloroplasts.

In comparison with previous research on related genes, the function of RAS in regulating chloroplast RNA processing shows both similarities and differences. Similarly to some known PPR proteins involved in chloroplast development, RAS affects the RNA editing and splicing of specific sites; however, the specific sites and the overall impact on chloroplast function vary among different proteins. For example, *OsTHA8* encodes a novel member of the PPR protein family [23]. The inactivation of this gene leads to abnormal chloroplast development, characterized by a deficiency in the editing of the RNA sites *rps8* and *ndhB*, as well as impaired splicing of the RNA site *ycf3* [23]. In this study, mutations in RAS affected the RNA editing at the *rpl2*, *rps14*, and *ndhA* sites, as well as RNA splicing at the *rpl2*, *atpF*, and *ndhA* transcripts within the chloroplast. This indicates that although PPR proteins share some common functions in chloroplast RNA processing, each protein has its own unique role and regulatory mechanism.

The efficient transport of nucleus-encoded chloroplast proteins into the chloroplast is vital for proper chloroplast function. This transport mechanism is predominantly reliant on the TOC (translocon on the outer chloroplast membrane) and TIC (translocon on the inner chloroplast membrane) complexes [48,49]. Once inside the chloroplast stroma, proteins may require assistance from molecular chaperones and guidance proteins to reach the thylakoid membranes via specific transport pathways [49,50]. Chloroplast proteins, including mature light-harvesting chlorophyll *a*/*b*-binding proteins (LHCPs), are transported to the thylakoid membranes by the chloroplast signal recognition particle (cpSRP) pathway [35,48]. In rice, cpSRP components include two homologous OscpSRPs, OscpSRP54a and OscpSRP54b, along with OscpSRP43. Mutants deficient in these cpSRP proteins exhibit impaired chloroplasts, reduced chlorophyll content, and regressed green leaves [36,37]. Similar phenotypes have been observed in cpSRP mutants of *Arabidopsis*, which display abnormal chloroplast development [35,51].

Both OsTRX z and RAS are encoded by genes located in the nucleus, and after being translated they are moved to the cytoplasm before being transported into the chloroplast. Previous studies have shown that OsTRX z and RAS influence PEP activity [4,5,39]. In our study, Y2H and BiFC assays demonstrated that OsTRX z and RAS interact with OscpSRP54b and OscpSRP43 in the chloroplast (Figure 7). Based on these findings, we speculate that the transport, localization, or folding of OsTRX z and RAS proteins in the chloroplast may be regulated and facilitated by cpSRP54 and cpSRP43.

## 4. Materials and Methods

### 4.1. Plant Materials and Growth Conditions

For the targeted modification of the *RAS* gene in *japonica* rice (*Oryza sativa* L. Zhonghua 11), a guide DNA (gDNA) sequence (GTCATGGTTGCGTGTGGTCG) was precisely selected and integrated into the CRISPR/Cas9 vector. The pRAS-Cas9 vector was successfully introduced into the rice genome by using the *Agrobacterium*-mediated method. Homozygous mutants, identified through sequencing in the T1 generation, were selected for further experimentation. The rice plants were grown in an incubator under the growth conditions of a cycle of 14 h of light (300 μmol photons m^−2^ s^−1^) and 10 h of darkness at 30 °C. A list of primer sequences used in the vector construction can be found in Supplementary Table S2.

### 4.2. Pigment Quantification

To quantify chlorophyll in 20-day-old leaves, we employed a method involving solvent extraction. Leaf segments, approximately 1 cm in size, were immersed in 10 mL of 80% acetone and left to soak in darkness for 48 h. Subsequent analysis was conducted using an ultraviolet spectrophotometer (DU800, Beckman, Brea, CA, USA) to measure the optical density (OD) at wavelengths specific to each pigment: 662 nm for chlorophyll *a* (Chl *a*) and 646 nm for chlorophyll *b* (Chl *b*). Each sample was analyzed in triplicate, with 80% acetone serving as the blank control [52].

### 4.3. Transmission Electron Microscopy (TEM) Assays

Leaf samples of 20-day-old WT and *ras* mutant plants were collected and processed for TEM analysis. The leaves were cut into small pieces. The samples were prepared according to standard procedures and analyzed with a Hitachi H-7650 electron microscope from Tokyo, Japan [53].

### 4.4. RNA Isolation and Quantitative Real-Time Polymerase Chain Reaction (qRT-PCR)

Total RNA was meticulously extracted from different rice tissue samples by using an RNAprep Pure Plant Kit (Tiangen, Beijing, China). Following this, complementary DNA (cDNA) synthesis was performed in accordance with the protocols outlined in the ReverTra Ace qPCR RT Master Mix Kit, which includes a genomic DNA remover (Toyobo, Osaka, Japan). qRT-PCR was performed using the LightCycler 96 System with a Toyobo SYBR Green Master Mix. The rice *UBQ5* gene was used as an internal control for normalization. A comprehensive list of primer sequences employed in the qRT-PCR can be found in Supplementary Table S2. Results are based on three independent biological replicates and are presented as means with their standard deviation (SD). Group comparisons were made by utilizing the Student’s *t*-test.

### 4.5. Subcellular Localization

In order to ascertain the intracellular localization of the RAS protein in rice, we utilized the primer pair RAS-GFP-F/R to amplify the entire coding sequence of *RAS*, omitting the stop codon. The transient expression GFP vector was then inserted with the amplified DNA fragment. The recombinant construct, designated as pRAS-GFP, along with the control vector GFP, was introduced into rice protoplasts. A laser scanning confocal microscope (LSM700, Carl Zeiss, Thornwood, NY, USA) was used to image the GFP fluorescence signal.

### 4.6. Chloroplast RNA Splicing and RNA Editing

The reverse transcription polymerase chain reaction (RT-PCR) was used to study the processing of chloroplast gene introns in both WT and *ras* mutant rice lines, distinguishing between group I and group II, according to established protocols [41]. The RT-PCR technique involved the use of targeted primers to amplify specific cDNA segments, which were subsequently subjected to analysis for identifying RNA editing events manifesting as C to T transitions [41]. A comprehensive list of primers deployed in the assessment of chloroplast RNA splicing and RNA editing is provided in Supplementary Table S2.

### 4.7. Yeast Two-Hybrid (Y2H) and Bimolecular Fluorescence Complementation (BiFC)

The coding sequence of *OsTRX z* was inserted into the pGBKT7 vector to generate a bait construct, while the *RAS* sequence was cloned into the pGADT7 vector to create a prey construct. These constructs were co-transformed in yeast and grown for 72 h at 30 °C on a selective medium lacking leucine and tryptophan. Positive interactions were recognized by the capacity of yeast colonies to thrive in a medium that did not contain leucine, tryptophan, histidine, and adenine.

Additionally, the coding sequences of *RAS* and *OsTRX z* were inserted into the 2YN vector, as appropriate, thereby generating the pRAC-2YN and pOsTRX z-2YN fusion vectors. The coding sequences for *RAS*, *OscpSRP54b*, and *OscpSRP43* were inserted into the 2YC vector, as appropriate, to establish the pRAC-2YC, pOscpSRP54b-2YC, and pOscpSRP43-2YC fusion vectors. The fusion vectors were grouped and introduced into rice protoplasts. A laser scanning confocal microscope (LSM700, Carl Zeiss, Thornwood, NY, USA) was employed for the observation of the transformed protoplasts.

The Y2H and BiFC assays were conducted in accordance with the methodologies previously described in earlier studies [4].

## 5. Conclusions

In conclusion, this study reveals that the pentatricopeptide repeat protein RAS is a key regulator in chloroplast development. Through its interaction with OsTRX z, it participates in essential RNA processing steps, such as the splicing and editing of specific chloroplast transcripts (*rpl2*, *rps14*, *ndhA*, and *atpF*), thereby influencing the expression of genes related to chloroplast formation, ribosome biogenesis, and chlorophyll biosynthesis. Moreover, the dependence of RAS and OsTRX z on the cpSRP pathway for proper localization within the chloroplast further emphasizes their significance in the complex regulatory machinery of chloroplast function. Our findings provide novel insights into the molecular mechanisms underlying chloroplast development and expand the understanding of the roles of PPR proteins in plants.

## Data Availability

Data are contained within the article and Supplementary Materials.

## Supplementary Materials

The following supporting information can be downloaded at https://www.mdpi.com/article/10.3390/plants14020247/s1: Figure S1. Sequence analysis of RAS. Figure S2. Splicing analysis of chloroplast transcripts in WT and *ras*. Table S1. Analysis of chloroplast RNA editing (C to U) in the WT and *ras* mutant. Table S2. List of primer pairs used in this study.
