# Supplementary material for: RAS, a Pentatricopeptide Repeat Protein, Interacts with OsTRX z to Regulate Chloroplast Gene Transcription and RNA Processing
Source: Plants (Basel). 2025 Jan 16;14(2):247. doi: 10.3390/plants14020247 (PMC11768195; doi:10.3390/plants14020247)
Supplement: Supplementary file 1 [file plants-14-00247-s001.zip › plants-3419187-supplementary.pdf]

Supplemental Figures

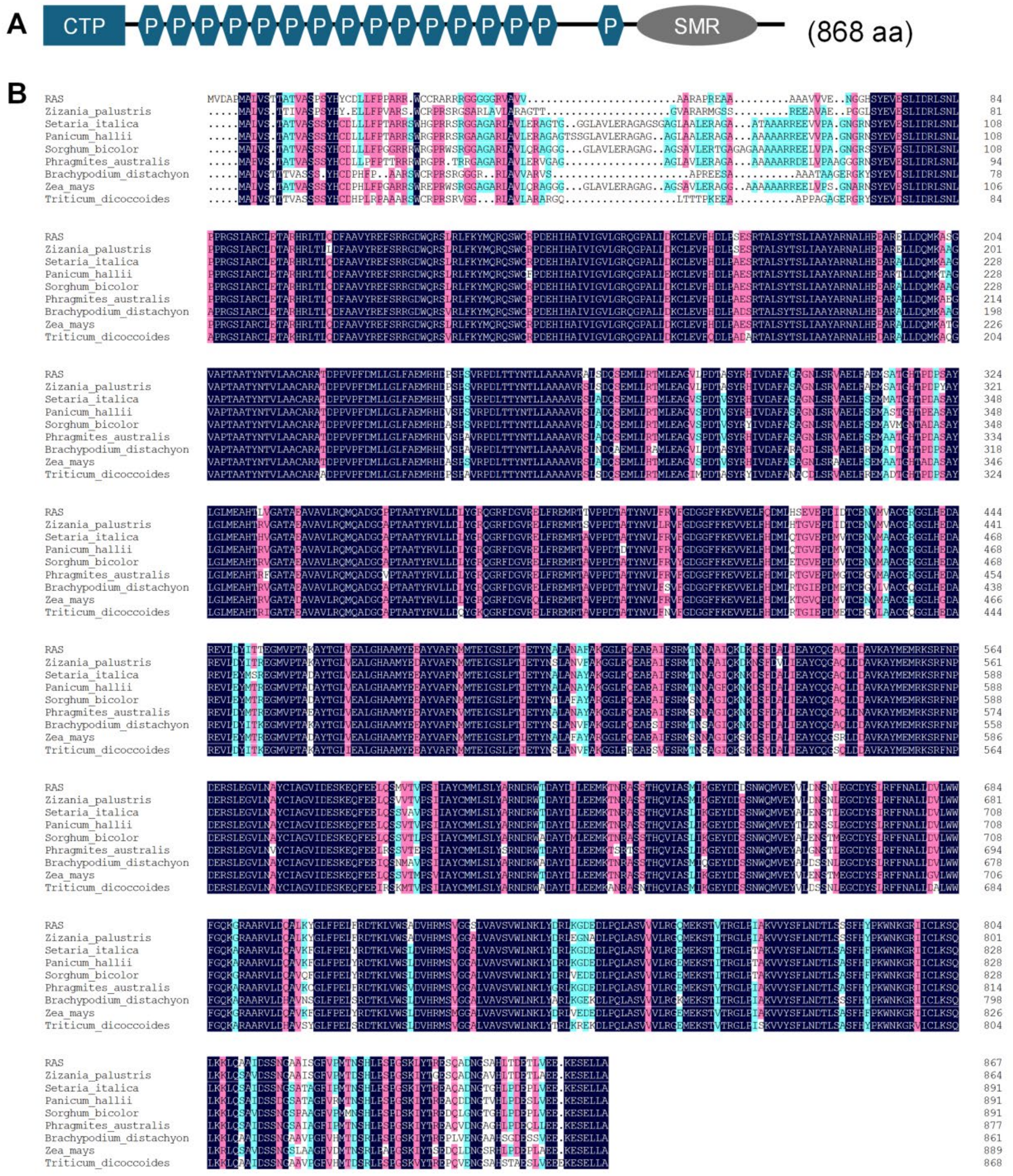

Figure S1. Sequence analysis of RAS

(**A**) Diagrams of the RAS sequence. The RAS protein contains 16 PPR motifs, an N-terminal chloroplast signal peptide (CTP) and a unique C-terminal SMR domain. (B) Amino acid sequence alignment of RAS homologs. Fully conserved amino acids are shaded in black, and partially conserved amino acids are shaded in gray. *Zizania palustris*, KAG8061576.1; *Setaria italica*, XP_004981223.2; *Panicum hallii*, XP_025793213.1; *Sorghum bicolor*, >XP_002463612.2; *Phragmites australis*, >XP_062212875.1; *Brachypodium distachyon*, >XP_003563463.1; *Zea mays*, >NP_001315315.1; *Triticum dicoccoides*, >XP_037436927.1.

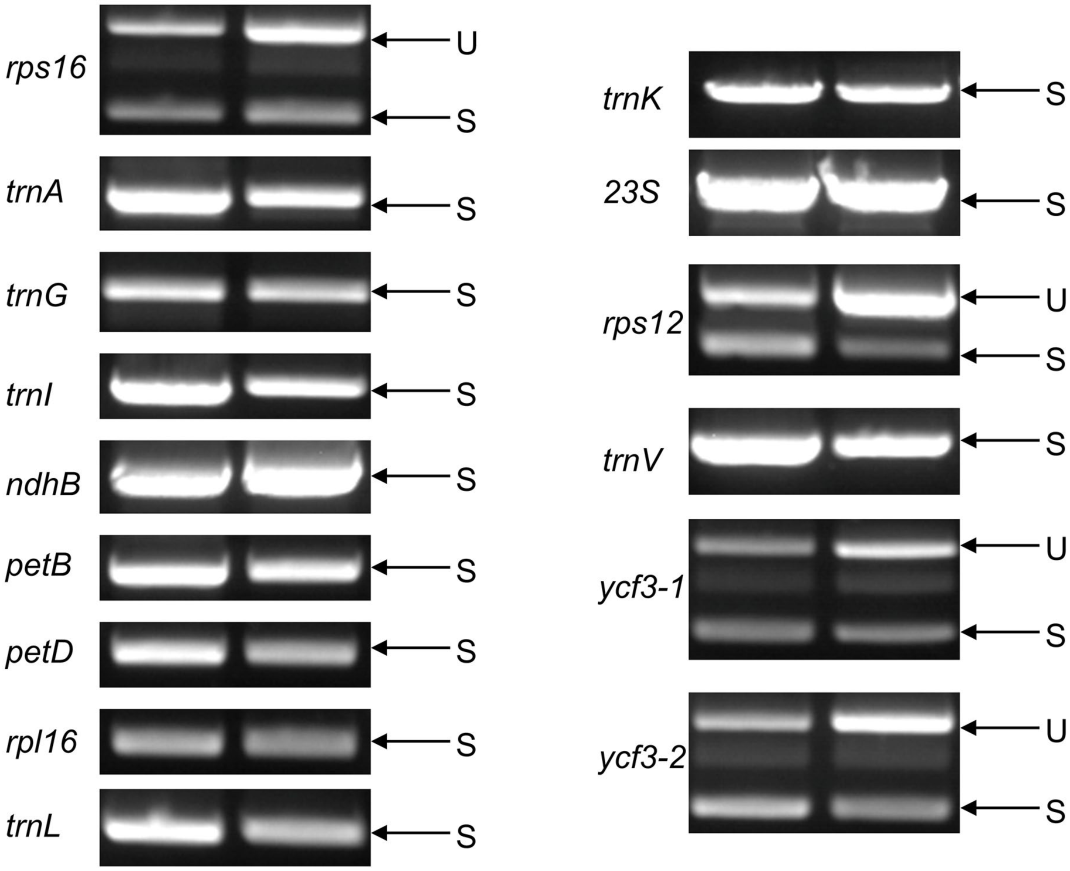

Figure S2. Splicing analysis of chloroplast transcripts in WT and *ras*

Unspliced (U) and Spliced (S) transcripts are shown on the right.

Supplemental Tables

Table S1. Analysis of chloroplast RNA editing (C to U) in the WT and *ras* mutant.

Gene	Editing position	Edited codon	Amino acid change	Transfer C to U (Y/N)	
				WT	*ras*
atpA	C1148	uCa	S→L	Y	Y
ndhA	C473	uCa	S→L	Y	N
	C563	uCa	S→L	N	N
	C1070	uCc	S→F	N	N
ndhB	C467	cCa	P→L	Y	Y
	C586	Cau	H→Y	Y	Y
	C611	uCa	S→L	Y	Y
	C704	uCc	S→F	Y	Y
	C737	cCa	P→L	Y	Y
	C830	uCa	S→L	Y	Y
	C836	uCa	S→L	Y	Y
	C1481	cCa	P→L	Y	Y
ndhD	C878	uCa	S→L	Y	Y
ndhF	C62	uCa	S→L	Y	Y
ndhG	C-10	5′-UTR	C→U	Y	Y
	C347	cCa	P→L	Y	Y
rpl2	C2	aCg	T→M	Y	N
rpoB	C467	uCg	S→L	Y	Y
	C545	uCa	S→L	Y	Y
	C560	uCa	S→L	Y	Y
rps8	C182	uCa	S→L	Y	Y
rps14	C80	uCa	S→L	Y	N
ycf3	C185	aCg	T→M	Y	Y

Table S2. List of primer pairs used in this study.

**Plasmid construction**		
Gene	Forward sequence (5′-3′)	Reverse sequence (5′-3′)
pRAS-Cas9	gcaGTCATGGTTGCGTGTGGTCGgttttag agctagaaatagcaagttaaaataag	CGACCACACGCAACCATGACtgcaccagc cgggaatcgaac
pRAS-GFP	TATTTACAATTACAGTCGACATGGTG GATGCTCCCATGG	ATGGATCCTCTAGAGTCGACCAGTGC AAGGAGTTCTGATTC
pOsTRX z-BD	ATGGAGGCCGAATTCATGGCCATGGC CGCGGCC	GATCCCCGGGAATTCTCACAATTCATT ATCAATGATATTTCTGA
pRAS-AD	gccatggaggccagtgaattcATGGTGGATGCT CCCATGG	atgcccacccgggtggaattcTCACAGTGCAAG GAGTTCTGATTC
pRAS-BD	TATTTACAATTACAGTCGACATGGTG	ATGGATCCTCTAGAGTCGACCAGTGC

	GATGCTCCCATGG	AAGGAGTTCTGATTC
pOscpSRP5<br>4b-AD	gaggccagtgaattcATGGAGGCCACCAGT<br>AGTACTACAC	gaggccagtgaattcTCATCGTCGACGGAAA<br>CCG
pOscpSRP4<br>3-AD	gccatggaggccagtgaattcATGGAGGCTGTC<br>CTACGACACC	atgcccacccgggtggaattcTCACCCGGCGAC<br>CGGCGG
pOsTRX<br>z-2YN	aattaaggcgcgccactagtATGGCCATGGCC<br>GCGGCC	ctgccacctcctccactagtCAATTCATTATCAA<br>TGATATTTCTGATCA
pRAS-2YN	aattaaggcgcgccactagtATGGTGGATGCTC<br>CCATGG	ctgccacctcctccactagtCAGTGCAAGGAGTT<br>CTGATTCCT
pRAS-2YC	aattaaggcgcgccactagtATGGTGGATGCTC<br>CCATGG	ctgccacctcctccactagtCAGTGCAAGGAGTT<br>CTGATTCCT
pOscpSRP5<br>4b-2YC	aattaaggcgcgccactagtATGGAGGCCACC<br>AGTAGTACTACAC	ctgccacctcctccactagtTCGTCGACGGAAAC<br>CGCG
pOscpSRP4<br>3-2YC	aattaaggcgcgccactagtATGGAGGCTGTC<br>CTACGACACC	ctgccacctcctccactagtCCCGGCGACCGGCG<br>GCGG
**qRT-PCR**		
Gene	Forward sequence (5′-3′)	Reverse sequence (5′-3′)
AtpB	TGAGAGGAATGGAAGTGATTGACA	TCAACAGGCTCCCCAAGAAC
AtpE	CGGTTCTGTGGAGCGGTTT	TGAGCTTCTTCCGGATCAATG
psaA	GTTTTCGCGGAGGGCTAGAT	TGACCTGCGATCAGGAAAAGA
psbA	ACTAGCACCGAAAACCGTCTTT	CAGCGATGAAGGCGATAATAAA
rbcL	CTCGCGGTATCTTTTTCACTCA	TCGGTCAGAGCTGGCATATG
RpoA	CGCATCAATTTGCGTCAAAG	GTTAGCTATAGGTTGTGCCGTATCAA
rpoB	CAAGTTTTCGGAGCCGAGAT	GCTAAAGATCCAGTAAGTCCAACGT
rpoC1	TCCGTCGGAACAACAATCTTG	TCCACGGCTTCTTGTACCAAT
rpoC2	ATGCATCGCAGGTACACCAA	CCCTCGCGTAAATTGCTTTG
HEMA1	GAACTCACCAGTCTGAATCATATTGA	CATCCAGTCTACCACTTCTCTAATCC
CHLI	GTTCGAGCCTGGTTTGCTTGC	CTCTCCACGGTGTTCCATCCTG
PORA	TGTACTGGAGCTGGAACAACAACT	TCAATAGCACATCACTCTCACTCACT
DVR	CAGGTCGAGACCGTCAAGAAC	ATGACCTGGATCGGCACCTTG
YGL1	GATAGAGCTCTGGGGCTTCAGTC	GCTTGCCGGAACTGAAAAGGTAG
CAO1	GACACCTTCATCTGGGCTTCAA	CGAGAGACATCCGGTAGAGC
16S rRNA	CCGTTGGTGTTCTTTCCGAT	TTCAAGTCCGCCGTCAAATC
23S rRNA	TGTGGGCGTTAGAGCATTGAG	CACTTGGCTACCCAGCGTTTA
rps4	CGATTAGGTATGGCTTCAAC	GATCTTTGGTTATCCTTCGTAG
rps11	GGTGCTGGTAGTGGAAGAGAT	GCGGCATAGGTGTTACATCG
rps12	AGCCGTTTGCTACCAATGG	TGATCGGTACCAATGAATAGG
rps14	GTTTGATTCAGAGAGAGAGG	TTCTCGAAGTATGTGTCCGG
rps16	CCTCGCGACAGACGTCCTAT	CTCCTCGTTAGGTGCTCCATC
rps18	CAACCTTTTCGCAAACCCAA	ATAATCAATTCGATCCCCCG
rpl16	ATGCTTAGTCCCAAAAGAACCAG	GCCTCGCCGTAATCCAAGT
rpl2	GGTACAAGCACCAGAAGGAG	GACAATCTTCTTGGCACCAGAG
UBQ5	CTCGCCGACTACAACATCCA	TCTTGGGCTTGGTGTACGTCTT

**RNA splicing**		
Gene	Forward sequence (5′-3′)	Reverse sequence (5′-3′)
atpF	ATGAAAAATGTAACCCATTCTT	TTCATCGCCCTTTGTTTTTC
ndhA	ATGATAATAGACAGGGTACAGG	TTATAGTGAAACAAGTTGGGAAG
ndhB	ATGATCTGGCATGTACAGAATG	CTAAAAGAGGGTATCCTGAGCA
petB	TTCTCATATACGGTTCTCGG	TAAAGGGCCCGAAATACCTT
petD	ATGGGAGTAACAAAGAAACC	TGTTGCTCCAATACCTAACC
rpl2	ACGGCGAAACATTTATACAA	TTACTTACGGCGACGAAGAATA
rpl16	ATGCTTAGTCCCAAAAGAAC	AACCGAAGAAATTGACTTCG
rps16	AAAACGATGTGGTAGAAAGC	AGAATTCCGCCTTCCTTAAA
trnA	GGGGATATAGCTCAGTTGGT	TGGAGATAAGCGGACTCGAA
trnG	TCGTTAGCTTGGAAGGCTAG	GCGGGTATAGTTTAGTGGTA
trnI	TGGGCCATCCTGGACTTGA	AGCTCAGTGGTAGAGCGCG
trnK	GGTTGCCCGGGACTCGAA	GGGTTGCTAACTCAATGGTAGAG
trnL	GGATATGGCGAAATCGGTA	TGGGGATAGAGGGACTTGA
trnV	TAGGGCTATACGGATTCGAA	AGGGCTATAGCTCAGTTCGG
ycf3-1	TGATAAGACCTTCTCAATTGTAGCC	GTGTGTATAAGGCCTATGTTATAGAGT
ycf3-2	AGAGCATACAAAGGCTTTGGAAT	TTCAACCAGTTCTGTGCTTCAATATA
rps12	ACTATCAACCCCAAAAAACC	TTTGGCTTTTTGACCCCAT
23S	TTCAAAAGAGGAAAGGCTTG	AGAGAGCACTCATCTTGGGG
**RNA editing**		
Gene	Forward sequence (5′-3′)	Reverse sequence (5′-3′)
ndhA	GGACCGTCTATAGCAGTCAT	TTTCTCTTGTTTGAGAGGAC
ndhB-1	ATGATCTGGCATGTACAGAATG	CTAAAAGAGGGTATCCTGAGCA
ndhB-2	CTTGGTTTCAATAGGACTCC	TAAAAGAGGGTATCCTGAGC
ndhD	ATTTTGGCTTCCTTATTGC	GCCTCTACCCTGTCAACG
ndhF	ATATGCATGGGTAATCCCTC	AGTGGCTCCTAAGAAAAGTG
ndhG	CCTAATCCCTTTTTTCTTCC	TCAAGACATTTATAGCTCCC
rpl2	CCGGGTTATTCTATTCCACT	TACGCATTTCGATTAGGGTC
rps8	ATGGGCAAGGACACTATTG	AACATAAGACTTCTCCCCCA
rps14	ATGGCAAAAAAAAGTTTGATTC	TTACCAACTGGATCTTGTTGCA
rpoB	GTCCTGSDGTATTTACTACCGC	TCCCCACCTACACAAGCAAA
atpA	CCCAGGGGATGTTTTTTATT	TGAAAAAAGCGTCCATTGTG
ycf3	ATGCCTAGATCCCGTATAAATG	TGTGGTAAGAAGGGGTTTCG
rpoC2	GGTCCTTGGGGATTCTTGAT	TCTTGTTTTGTGGGTAACGG
